# The four of structural genes sequences of a porcine epidemic diarrhea virus from domestic piglet in Fujian, China

**DOI:** 10.1186/s12985-020-01345-7

**Published:** 2020-06-18

**Authors:** Bo Dong, Ailing Dai, Xiaohua Li, Xiaoyan Yang

**Affiliations:** 1 College of Life Science of Longyan University, Longyan, 364012 China; 2 Fujian Provincial Key Laboratory for the Prevention and Control of Animal Infectious Diseases and Biotechnology, Longyan, 364012 China

**Keywords:** Porcine epidemic diarrhea virus, Spike gene, Small membrane gene, Membrane gene, Nucleocapsid gene, Phylogenetic analysis, Genetic characterization

## Abstract

A prevalent PEDV strain, designated FJLY06, was isolated from Fujian, China. The four of structural genes sequences of PEDV obtained were analyzed to determine their phylogenetic relationships and homology respectively, revealing that FJLY06 was highly homologous to virulent PEDV strains. The four structural genes all differed genetically from the vaccine strain CV777. The sequence alignment results further showed that N, M and E genes of Chinese PEDV strains is highly conserved. Compared with the vaccine strain CV777, 8 mutations were detected in COE of FJLY06 S gene. The recombination analysis revealed FJLY06 is similar to other pandemic strains in China with a variant S gene, and maybe a reason for recent vaccination failures.

## Introduction

PEDV has four major structural proteins: spike (S), envelope (E), membrane (M), and nucleocapsid (N) proteins [1]. Since the 1990s, a periodic vaccination strategy has been placed into effect on pig farms to control PEDV in China. However, these vaccines were not able to prevent the disease effectively in 2010. This suggests that the virulence of pandemic strains has become stronger, and might possibly provide protection to the CV777-based vaccine, thereby reducing the effectiveness of the vaccine. Analysis of a single structural gene may not really indicate genetic evolution because it reflects only a portion of the virus’s genes, in contrast, using four structural genes may be eliminate the bias of using a single gene for genetic phylogenetic analysis in PEDV. A clear understanding of relevant epidemiological parameters is a key to planning better treatment and control strategies.

The sample was obtained from sucking piglets showing watery diarrhea and dehydration, which could not be cured with any commercial available antibiotic. The E, M, N and S genes of this PEDV strain was cloned and sequenced to investigate the genetic characteristics and phylogeny of PEDV circulating in Fujian during this period. These data will provide a basis for further analysis of the genetic evolution of PEDV in China, and should help to develop a novel vaccine of PEDV for more effective prevention piglets from PEDV.

### Provenance of the virus material

The FJLY06 was isolated from a sucking piglet collected in the Fujian province of China. After the piglet was necropsied, tissue samples of the intestinal, feces and intestinal contents from piglet were homogenized and 10% (w/v) suspensions were made in sterile Dulbecco’s phosphate-buffered saline (PBS, pH 7.2, 0.01 M). RNA was extracted from the sample using RNAiso Plus (TaKaRa, Tokyo, Japan). Reverse transcription was carried out and PCR was performed (Biometra, Germany) using the gene-specific primers. The amplified PCR products were subjected to gel electrophoresis, excised from the agarose gel, and purified using an Agarose Gel DNA Purification Kit (TaKaRa). The clones were sent to Shanghai Sangon Biological Engineering Technology and Services Co., Ltd. (Shanghai, China) to be sequenced. The S, E, M and N gene sequences of the FJLY06 were edited, aligned and analyzed with the DNAMAN software. The nucleotides sequences were assembled into a multiple sequence alignment. Phylogenetic trees derived from the nucleotides sequences were constructed using the program MEGA version 5.2 [2]. The SimPlot 3.5 was used for similarity mapping and bootscanning analysis of potential reorganization events.

### Sequence properties

Compared with the nucleotides sequences of 51 strains used in our study, the N gene of FJLY06 showed nucleotide identities of 94.3% to the vaccine strain CV777 used in China. Sequence comparison with the CV777 revealed that E gene of FJLY06 had nucleotide sequence identities of 96.5%. The M gene had 98.7% identity to the CV777 vaccine strain. The nucleotide sequence homology results of S gene showed FJLY06 had the low DNA sequence identity to the CV777, which is 92.2%.

**Fig. 1:**
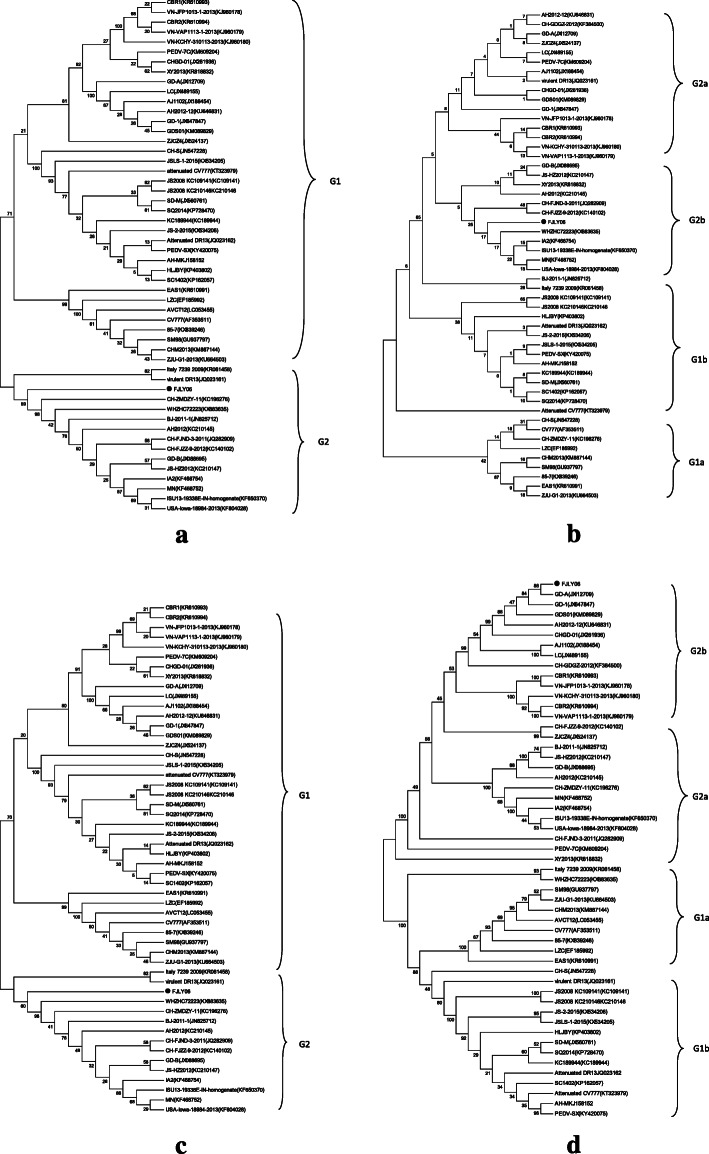
Phylogenetic trees were constructed using MEGA 5.2 software based on comparisons of N, E, M and S nucleotide sequences. **a** Tree based on nucleotide sequences of N gene. **b** Tree based on nucleotide sequences of E gene. **c** Tree based on nucleotide sequences of M gene. **d** Tree based on nucleotide sequences of S gene

The phylogenetic analysis found that the evolutionary relationship of N, M, E and S genes of the FJLY06 from our study were more closely with Chinese strains isolated after 2010, and belong to the PEDV strain variants. Meanwhile, the results showed that the FJLY06 from our study differed genetically from the vaccine strain (CV777) and other earlier PEDV strains found in China, South Korea and Belgium, as well as some classical strains (Fig. 1.)

Interesting, phylogenetic analysis showed that FJLY06 was located in the same subgroup with GD-01 collected previously from the Guangdong province of China [3], which is neighboring province of Fujian province, where FJLY06 is circulating. This result suggested whether animal transportation could be a risk factor in the spread of PEDV, because there was a relatively frequent flow of pigs or pork products between this two provinces in China.

Compared with the N gene of vaccine strain CV777, 12 mutations were detected in the amino acid sequence of the FJLY06 (G → A at 84, K → N at 123, A → T at 142, N → K at 204, R → K at 241, H → L at 242, K → R at 252, N → S at 255, L → P at 380, L → Q at 395, Q → L at 397, and E → D at 400). There was no difference compared to those identified in Chinese strains isolated after 2010. In terms of amino acid sequence of M genes, the G2 including the FJLY06 and other prevalent variant strains had the same three unique mutations as CV777, (E → Q at 12, A → V at 42, and A → S at 213), and no difference was found in the M gene of the FJLY06 when compared to the other prevalent variant strains. It also had a unique point mutation in E gene of the FJLY06 compared with CV777 (R → Q at 65), which was similar to the other strains of G2 group.

The S gene of PEDV contains four domains that can induce neutralizing antibodies, COE (499–638 aa), SS2 (748–755 aa), SS6 (764–771 aa), and 2C10 (1368–1374 aa) [4]. The FJLY06 had 8 mutations compared to COE of the classical vaccine strain CV777 (I → H at 521, S → G at 523, V → I at 527, T → S at 550, G → S at 594, A → E at 605, L → F at 612, and I → V at 635). In the other three neutralizing epitopes compared with CV777, there was only one mutation in the other three neutralizing epitopes (Y → S at 766, in SS6), suggesting that CV777-based vaccines are not effective to prevent and control PEDV epidemics, indicating the need to develop and select new vaccines.

**Fig. 2:**
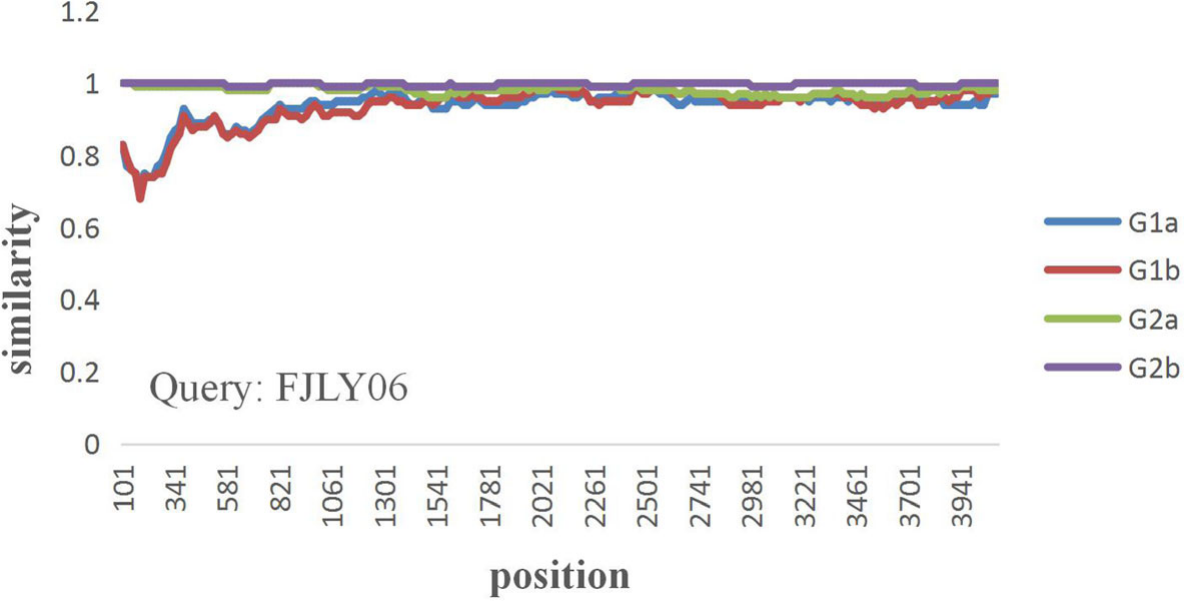
Detection of potential recombination events in FJLY06. SimPlot analysis for possible recombination events of S genes of Fujian’s strains compared to the G1 and G2 groups

Nucleotide sequence similarity were assessed by SimPlotv.3.5.1, and showed that the FJLY06 is very similar to other sequences in G2 group (Fig. 1d) and can be inferred from the same ancestor. However, there were differences between Fujian’s strains and reference strains in G1 group (Fig. 1d), located within N-terminus of the S protein (Fig. 2), suggested this region in the S gene exhibited an increased divergence compared to the remaining part of the sequence, and the recently isolated prevailing PEDV strain in Fujian is a novel strains with a variant S gene and maybe originated between classical and variant strains [5, 6].

## Data Availability

The data analyzed during the current study was available from the corresponding author on reasonable request.

## Abbreviations

PEDV: Porcine epidemic diarrhea virus
